# California State Veterinary Medical Association

**Published:** 1895-04

**Authors:** R. A. Archibald

**Affiliations:** Secretary


### SOCIETY PROCEEDINGS.

### CALIFORNIA STATE VETERINARY MEDICAL ASSOCIATION.

The California State Veterinary Medical Association held its regular quarterly meeting at the St. James Hotel, San José, Cal., March 13, 1895.

The President, Dr. C. B. Orvis, called the meeting to order at 3 P.M.

Upon roll-call the following members were found to be present: Drs. Orvis, Spencer, Sr., Spencer, Jr., Egan, Forrest, Sr., Forrest, Jr., Wadams, Schodde, Shaw, Skaife, Fabbi, and Archibald. Visitor, Prof. A. A. Cunningham.

Letters of regret were read from Drs. Pierce and Maclay.

Under the head of reports of Board of Examiners, Committees, etc., there not being a quorum of the Board of Examiners present, the Chair appointed the Secretary to act on the Board in conjunction with the two members of the Board that were present. A recess was declared in order to allow the Board to consider applications for membership. Upon the meeting reconvening the Secretary submitted the following report: Mr. President and Gentlemen: Your Board of Examiners having had under consideration the applications of Drs. J. R. Shaw, A. B. Wise, J. A. Edmons, J. J. Streets, B. W. Schodde, J. W. O'Rourke, E. J. Creely, S. A. Withers, and R. A. Withers, respectfully recommend that they be elected to membership; also having had under consideration the applications of O. C. Baldy and J. Trullinger, respectfully recommend that their application be rejected. Signed by Drs. Spencer, Sr., Egan, and Archibald. On motion, duly seconded, the report was accepted and placed on file.

The Committee on Legislation was then called upon to report. Owing, however, to the absence of the Chairman, Dr. Spencer, Sr., gave a partial report which was completed by the Secretary. The report was accepted and the Committee discharged. In this connection Dr. Spencer, Sr., made a few remarks regarding the support given the Committee on Legislation by the members of the Association. He stated that the failure on the part of the Committee to obtain the passage of Assembly Bill No. 210 was entirely due to the lack of interest taken in the matter by the members. The Secretary also made a few remarks on this subject, in which he stated that it was impossible for one or two members of the Association to obtain any legislation alone; he also stated that where a legislator had been interviewed by a veterinarian from his district he invariably proved a friend to any bill introduced by the profession.

Under the head of Application for Membership, the Secretary presented the name of C. L. Megowan. The name was referred to the Board of Examiners. Under the head of Admission of New Members, the following gentlemen were elected: Drs. J. R. Shaw, A. B. Wise, J. A. Edmons, J. J. Streets, W. B. Schodde, J. W. O'Rourke, S. A. Withers, J. A. Withers, and E. J. Creely.

Under the head of New Business, Dr. Wadams moved that the thanks of the Association be tendered Assemblyman Berry for the great interest he had taken in Assembly Bill No. 210. Carried.

On motion by the Secretary, a committee consisting of Drs. Wadams and Spencer, Sr., were appointed to convey the thanks of the Association to Assemblyman Berry in a suitable manner.

The subject matter of sending a delegate to the next meeting of the United States Veterinary Medical Association was brought up by Dr. R. A. Archibald, who endeavored to portray to the members the necessity of such action on their part. He also stated that it was his opinion that if the Association evinced sufficient interest in the matter during the next two years that the United States Veterinary Medical Association would decide upon California as the place of meeting in the year 1897.

Dr. Spencer, Sr., also spoke on this subject in the same strain as the Secretary had done.

The Secretary moved that the Association levy an assessment of five dollars per capita for the purpose of defraying expenses of a delegate to the next meeting of the United States Veterinary Medical Association, also that the Secretary be instructed to collect same, and at the same time notify the members that at the next quarterly meeting, held in June, a delegate would be elected. The motion was duly seconded, and after considerable favorable discussion it was eventually adopted unanimously.

Prof. W. F. Skaife, in behalf of the Faculty of the California Veterinary College, extended to the Association an invitation to hold their meeting in the college building when they again meet in San Francisco.

The following resolutions were presented and adopted unanimously:

*Whereas*, It is a notorious fact that during the twenty-ninth, thirtieth, and thirty-first sessions of the State Legislature, one Thomas Carpenter, a veterinary surgeon and a resident of Oakland, did use all means in his power to defeat any and all movements toward the advancement of the veterinary profession in the State; and,

*Whereas*, This action on the part of said Thomas Carpenter was wholly due to the jealous and abject sentiment which rankled in his breast toward the members of the California State Veterinary Medical Association and other members of the veterinary profession in the State, who have always and are yet struggling to gain public recognition as practical sanitarians and as scientific gentlemen; and,

*Whereas*, The veterinary profession in the State of California has suffered through the actions and machinations of the said Thomas Carpenter; and,

*Whereas*, The said Thomas Carpenter is at the present time endeavoring to gain the position of Meat and Milk Inspector to the Board of Health of the city of Oakland; therefore be it

*Resolved*, That we, the members of the California State Veterinary Medical Association, do hereby protest against the appointment of the said Thomas Carpenter to the office of Meat and Milk Inspector in the city of Oakland, as we believe that such action on the part of the health authorities would work a hardship on the people of the city of Oakland and at the same time be a detriment to the interests of the veterinary profession throughout the State of California; and be it further

*Resolved*, That a copy of these resolutions be forwarded by the Secretary to the Oakland Board of Health and that a copy be spread upon the minutes.

Upon motion, the President, Dr. C. B. Orvis, was authorized to represent the Association at the next meeting of the State Sanitary Convention, to be held in San Francisco in April.

The Secretary moved that when this meeting adjourns it adjourns to meet in Stockton, on Wednesday, June 12, 1895.

Upon motion, the Association took a recess until 8 P.M.

*Evening Session.* The Association reconvened at 8 P.M., President Orvis in the chair.

Under the head of Reading of Papers and Discussions, the President, Dr. C. B. Orvis, delivered his inaugural address, in which he thanked the members for the great honor they had conferred upon him. He reviewed the progress of the profession in this State in the past nine years, also the efforts of the profession to obtain suitable legislation. He dwelt considerably on the prevalence of glanders, tuberculosis, and other contagious and infectious animal diseases, and he deplored the lack of proper laws for the control of the same. The address was listened to with much interest by the members present, and the applause that followed its reading showed the manner in which it was appreciated.

The next business on the programme was the reading of a very interesting and instructive paper by Dr. A. H. Spencer, entitled "Antiseptic Surgery."[1] As usual, the Doctor submitted for the consideration of the mem-

[1] Will be published in May number.

bers a spicy, practical, and well-written thesis. The discussion that followed demonstrated the fact that the subject was well chosen, as the members to a man participated in the discussion and showed by their attention and interest that they considered it to be one of the most important subjects that could have been submitted to them.

Owing to the unavoidable absence of Drs. Faulkner and Jackson, who were also appointed essayists for this meeting, no more papers were presented, but several interesting cases were reported by the different members.

Upon motion, a vote of thanks was tendered Drs. Orvis and Spencer, Sr., for the able and zealous manner in which they had entertained the meeting.

The Chair appointed the following named gentlemen as essayists for the next meeting: Drs. Fox, Skaife, Eddy, and Fabbi.

The Secretary made his regular quarterly statement regarding the advisability of as many of the members as possible joining the U. S. V. M. A. He stated that he could supply application blanks to those who wished to make application for membership.

Drs. H. A. Spencer and H. F. Spencer extended to the members a cordial invitation to meet at their hospital and participate in a clinical entertainment; in behalf of the members the Chair accepted the invitation in the same spirit it was given.

There being no further business before the meeting, it adjourned to meet in Stockton on June 12, 1895.

*Clinical Entertainment.* On March 14, 1895, at 9 A.M., the members of the California State Veterinary Medical Association assembled at the veterinary hospital at 224 E. St. John Street, San José, on invitation of Drs. H. A. Spencer and H. F. Spencer, to participate in a clinical entertainment.

The first and second operations on the programme were the castration of a double cryptorchid and a single cryptorchid by Dr. H. A. Spencer. In the performance of these operations the Doctor demonstrated the fact that he has no superior as an operator in the United States.

The third operation on the programme was the extirpation of a champignon by Dr. Archibald, ably assisted by Dr. H. F. Spencer. The operator demonstrated his method of operating on these cases on the operating table. The tumor resected was quite large, and as far as its removal was concerned it was successfully accomplished.

The fourth operation on the programme was ovariotomy on a bitch, by Dr. Archibald. This proved to be a peculiar case, inasmuch as it was found, when the operation was well advanced, that the animal was pregnant; however, the operation was completed, and from the latest accounts the bitch seemed to be doing well.

The fifth operation was the application of the actual cautery for the cure of side-bones. This operation was demonstrated by Dr. J. R. Shaw, who performed the operation with the thermo-cautery. The members were loud in their praises of the manner in which the operation was performed.

The sixth operation was the removal of a number of hypertrophied lymphatic glands from the neck of a dog. This operation was performed by Dr. H. F. Spencer, who gave ample proof of his ability as a surgeon of no ordinary skill and ingenuity.

The seventh operation was the amputation of the leg of a fox-terrier bitch at the tibial region. This operation was performed by Drs. Spencer, Jr. and Archibald.

The members highly commended the manner in which the entertainment was provided for by the San José veterinarians, and upon the completion of the seventh and last operation they dispersed to their respective localities with the feeling that the time purloined from their business was by no means thrown away, and all expressed the conviction that clinical entertainments in connection with our meetings were a success, and that in future no ordinary circumstances would prevent them from being present at similar entertainments.

R. A. ARCHIBALD, V.S.,
Secretary.

